# A Card

**Published:** 1841-05

**Authors:** 


THE WESTERN JOURNAL.

Vol. III.—No. V.

LOUISVILLE, MAY 1, 1841.

A CARD.

Dr. Drake takes this method of informing those who may wish to address him on the subject of the Journal, or may desire his professional advice, that he will reside in Cincinnati, until the opening of the next session of the Medical Institute of Louisville, on the first of November.

